# Interpersonal relations psychotherapy for breast cancer patients: a systematic review

**DOI:** 10.1192/j.eurpsy.2025.1252

**Published:** 2025-08-26

**Authors:** T. Şahin Tokatlıoğlu, F. Oflaz

**Affiliations:** 1 Istanbul Aydın University, Faculty of Health Sciences; 2 Koç University, School of Nursing, İstanbul, Türkiye

## Abstract

**Introduction:**

Breast cancer (BC), one of the most common cancer types among women, affects one in four women according to global cancer statistics (Ferlay et al., 2021; Yusof et al., 2021; Sung et al. 2021; Li et al. 2023). Despite the increase in the life expectancy of cancer patients with advances in treatments and developing technology, its diagnosis as a disease with a high risk of death causes anxiety and stress in individuals (Solomon et al., 2000; WHO, 2018; Miller et al., 2019; Abdollahi et al., 2019).

In addition to physical and emotional changes, the diagnosis of cancer brings about changes in social relationships and roles. In coping with these processes, it has been reported that social support and social support management will have positive effects on coping with the problems brought by the disease, improvement in prognosis and self-management levels of patients (Xiang, 2010).

Interpersonal Relationship Psychotherapy (IRPT), which has been recommended as monotherapy for mild to moderate depression in treatment protocols in recent years (Spinelli, 1997; Spinelli and Endicott., 2003; APA, 2010; Brandom et al. 2012) Interpersonal psychotherapy has been found to be effective in the short and long-term treatment of cancer patients with major depressive disorder (Blanco, Hershman et al., 2014). Grief, interpersonal conflicts, role changes and social isolation (lack of interpersonal skills), which are the focus of IPT, are frequently experienced problems in cancer-related processes. IPT works by associating the social support and interpersonal problems that patients receive with disease symptoms (Sayar & Omay, 2019).

**Objectives:**

This study will examine the effect of Interpersonal Relationship Psychotherapy (IRPT), which is recommended as monotherapy for mild to moderate depression in treatment protocols, in breast cancer patients.

**Methods:**

Studies conducted in the last 10 years and in Turkish-English are included.

95 publications were reached. 73 publications were reached after removing duplicate publications.

We included randomized controlled trials of interpersonal relationship psychotherapy in breast cancer patients, comparing it with combination therapy, and applying it by telephone. The study included 4 studies.

**Results:**

Having examined a total of 73 abstracts, we retrieved 10 full-text papers for further study. Of these, we excluded 6 papers that did not meet inclusion criteria. A total of 4 studies met all inclusion criteria and were included in this systematic review.

**Image 1:**

**Figure:**
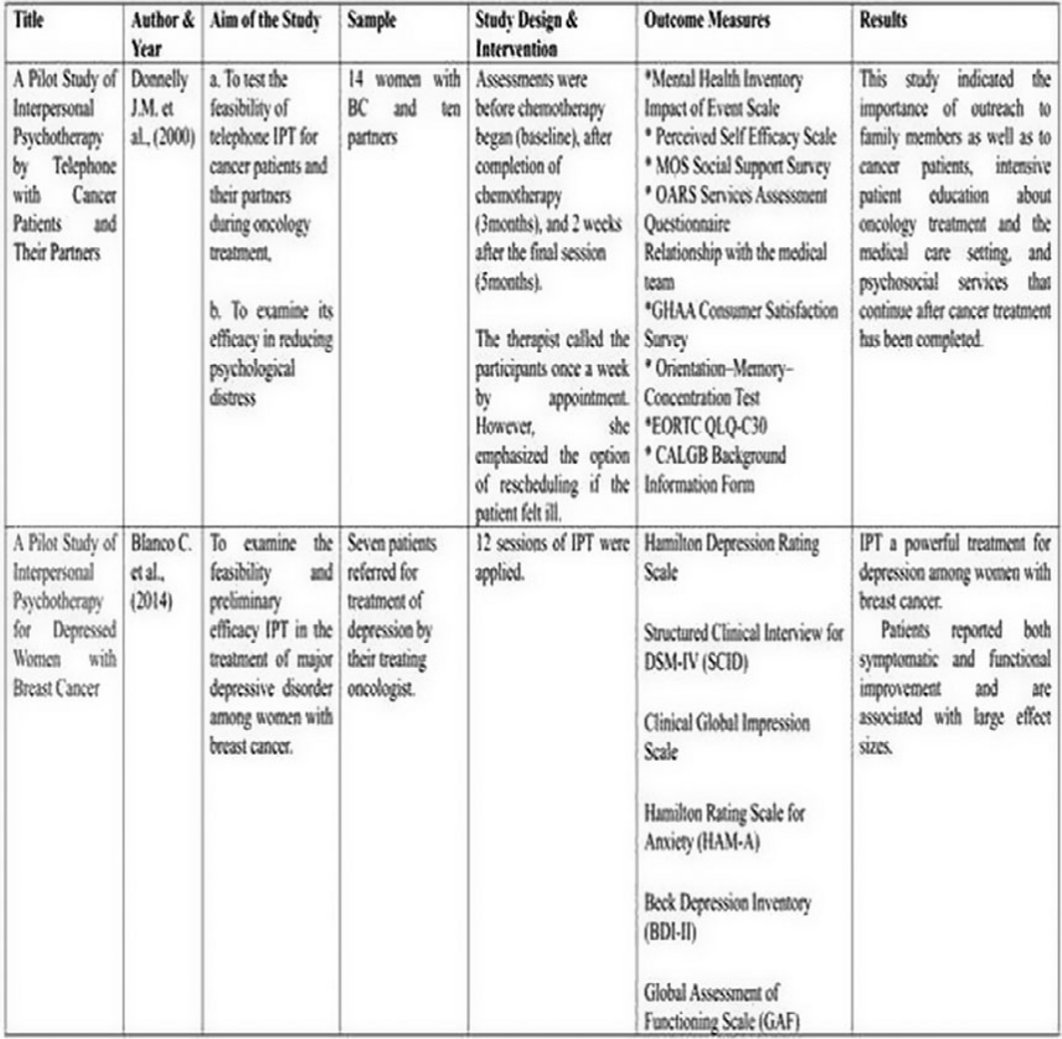

**Image 2:**

**Figure:**
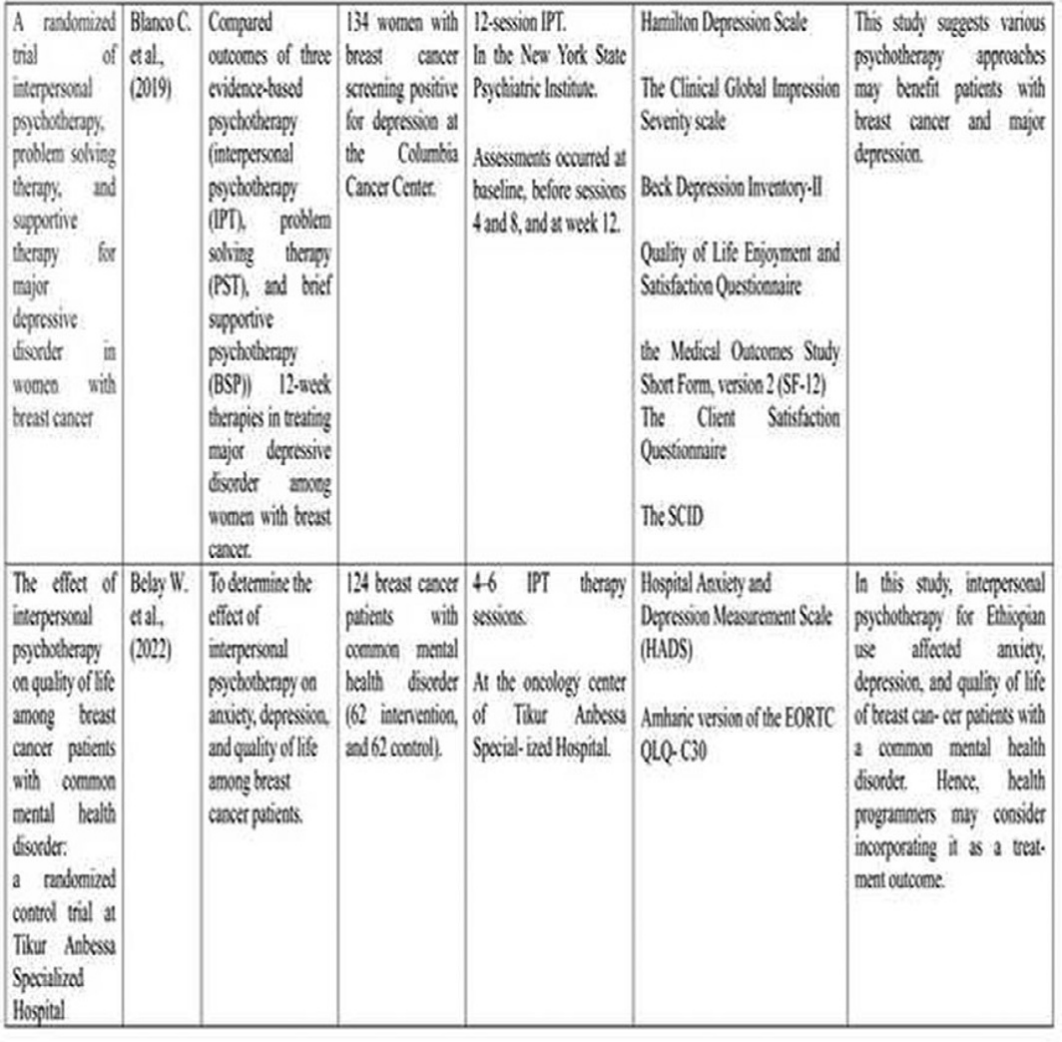

**Conclusions:**

When the results of four studies examining the effectiveness of interpersonal relationship psychotherapy were examined, it was found to have a positive effect for breast cancer patients. This study, which examines the effects of interpersonal relationship psychotherapy, a therapy with proven positive effects on depression in different samples, on breast cancer patients, is important because it provides evidence to the literature.

**Disclosure of Interest:**

None Declared

